# Calcium enriched mixture versus mineral trioxide aggregate in vital pulp therapy in primary teeth: a systematic review

**DOI:** 10.1097/MS9.0000000000004667

**Published:** 2026-01-26

**Authors:** Lferde Merieme, Bouziane Amal, Ramdi Hind

**Affiliations:** a Faculty of Dental Medicine, Mohammed V University in Rabat, Morocco; b Laboratory of Biostatistics, Clinical Research and Epidemiology, Faculty of Medicine and Pharmacy, Mohammed V University in Rabat, Morocco

## Abstract

**Aim:**

This systematic review aimed to assess the clinical and radiographic success of vital pulp therapy in primary teeth using calcium-enriched mixture (CEM) compared to mineral trioxide aggregate (MTA).

**Methods:**

Five electronic databases (PubMed, Scopus, EBSCO, Cochrane, and Web of Science) were searched for randomized controlled trials (RCTs) comparing the success rates of CEM and MTA as pulp dressing materials in primary teeth. The search strategy included terms related to CEM, vital pulp therapy, and primary teeth. The quality of the included studies was assessed using the revised risk of bias tool for randomized trials (RoB 2).

**Results:**

The search yielded 1834 publications, of which three RCTs met the inclusion criteria. These studies, published between 2011 and 2016, included a total of 108 children aged 4–8 years. All included studies were judged to raise some concerns regarding the risk of bias.

**Conclusions:**

Based on the limited available evidence, CEM may be considered a potential alternative to MTA for vital pulp therapy in primary teeth, with similar clinical and radiographic success rates. However, due to concerns about study quality and small sample sizes, further well-designed randomized clinical trials are necessary to draw more definitive conclusions.

## Introduction

The objective of vital pulp therapy (VPT) in primary teeth is to manage reversible pulpal injuries^[1]^. This procedure creates, through the use of an appropriate capping agent, a favorable environment that promotes pulp tissue healing and prevents potential bacterial invasion.

To this effect, many medicaments have been proposed, among them the mineral trioxide aggregate (MTA) and the calcium-enriched mixture (CEM).

MTA was released in 1993 to repair lateral root perforations and was granted approval for human use by the U.S. Food and Drug Administration in 1998^[2]^.

It has been successfully recommended for a wide variety of endodontic treatment^[3]^. However, it has several drawbacks such as its expensive cost^[4]^, its poor handling characteristics^[5]^, its short shelf-life since it is sensitive to moisture, and its delayed hardening time and the risk of tooth discoloration^[6]^.

**HIGHLIGHTS**

- The success rate of calcium-enriched mixture (CEM) in vital pulp therapy for primary teeth was found to be comparable to that of mineral trioxide aggregate (MTA).
- CEM emerges as a potential alternative to MTA for vital pulp therapy in primary teeth
- Further randomized clinical trials with larger sample size and extended follow-up periods are highly encouraged.

CEM is a more recent calcium-based cement, used as a root canal filling materiel in 2006 to overcome the drawbacks of MTA^[7]^. Studies have shown that, like MTA, CEM is a biocompatible and osteoinductive material^[8–10]^. It has been successfully tested in several studies for the same clinical applications as MTA^[11–14]^. Thus, considering that no systematic review has been published to present a high level of evidence on its use in primary teeth, this study is particularly important because of its clinical relevance.

Therefore, the objective of this systematic review was to answer the following research question: in randomized clinical trials, does the use of CEM as a pulp capping agent for VPT in primary teeth, compared to MTA, result in significantly higher clinical and radiographic success rates?

## Methods

### Reporting

The protocol was designed according to PRISMA (Preferred Reporting Items for Systematic Reviews and Meta-Analysis) guidelines^[15]^. Furthermore, it was preliminarily recorded in the International Prospective Register of Systematic Reviews (PROSPERO) database.

### Focused question

The research question was structured following PICOS (population, intervention, comparison, outcome, and study design) framework. In randomized clinical trials, does the use of CEM as a pulp capping agent for VPT in primary teeth, compared to MTA, lead to significantly higher clinical and radiographic success rates (Table 1)?

**Table 1:** Focused question

PICOS	Eligibility criteria
Population	Children with at least one primary tooth candidate for a vital pulp therapy and with no systemic disease
Intervention	Calcium-enriched mixture cement
Comparators	Mineral trioxide aggregate
Outcomes	Primary outcomes
	Clinical and radiographic success
	secondary outcomes
	Subjective evaluation of the patient and the practitioner: comfort, handling. Treatment costs: required time, materials, and price Adverse events: allergy toward materials used, tooth discoloration
Study design	Randomized controlled clinical trials

PICOS: **Population, intervention, comparator, outcomes, and study design.**

Primary and secondary endpoints:

The judgment criteria for assessing clinical and radiographic success are as follow:

- Clinical success is defined as the absence of pain, abscess or fistula, excessive tooth mobility or swelling.
- Radiographic success is defined as the absence of radiolucency (apical or furcal), root resorption (internal or external), periodontal ligament widening, or periapical bone destruction.

### Search strategy

Systematic search strategies were conducted by searching the following electronic databases: The Cochrane Central Register of Controlled Trials (CENTRAL), MEDLINE (PubMed), Scopus, CINAHL (EBSCO), Web of Science, and Clinical trials databases until the 2 October 2025 without any restrictions on language.

To maximize the identification of relevant studies, a search was conducted within the journal databases of the International Journal of Pediatric Dentistry and the European Journal of Paediatric Dentistry. In addition, other databases were searched as source of gray literature: OpenGrey (http://www.opengrey.eu/), Google Scholar, and conference’s abstract using manual searching. Furthermore, the ISRCTN registry (www.isrctn.com) and the EU Clinical Trials Register (www.clinicaltrialsregister.eu) were screened for appropriate ongoing or unpublished studies.

Manual searches of the reference sections of the included manuscripts were also conducted.

An initial search strategy was developed for MEDLINE using appropriate controlled vocabulary (Medical Subject Headings) and uncontrolled vocabulary (textual words and their synonyms) combined with Boolean operators (AND, OR). The equation was structured as follows:

(calcium enriched mixture cement OR Calcium-Enriched Mixture OR CEM Cement OR CEM OR Dental cements OR endodontic cement OR NEC OR calcium compounds OR bioceramic) AND (Dental Pulp Capping OR Pulpotomy OR Direct Pulp Capping OR Pulp Capping OR Vital Pulp Therapy OR Pulp exposure) AND (primary teeth OR primary molar OR deciduous tooth OR deciduous dentition)

Then, the search strategy was then specifically adjusted for each database (Supplemental Digital Content Appendix, available at: http://links.lww.com/MS9/B91).

### Data selection

Title and abstract of each article identified by our search strategies were independently examined by two reviewers to initially select potentially relevant studies (L.M. and R.H). Full-text versions were then obtained and categorized as either eligible or ineligible.

Disagreements between reviewers were resolved through discussion. If consensus could not be reached, a third reviewer was designated to make the final decision (B.A.). Reports that met all inclusion criteria were then used for data extraction.

#### Inclusion criteria

Included papers fulfilled the following criteria:

- Randomized controlled clinical trials (RCTs) comparing CEM outcomes vs MTA in VPT performed on primary teeth.
- Only studies including patients with no general health problem were considered to avoid any possible interference with the pulp healing process.

#### Exclusion criteria

Excluded papers fulfilled at least one of the following criteria: animal studies and studies using any other materials. Case reports, case series, *in vitro* studies, editorials, and review articles were excluded as well.

### Data extraction

All studies that fulfilled the inclusion criteria were subjected to data collection. This process was carried out independently by two authors, following a standardized data collection sheet. The sheet included all relevant variables necessary for interpreting the results, such as the subsequent items: first author/year of publication (country), study design, intervention procedure, sample characteristic, preoperative status of the teeth, restoration, isolation, assessment criteria, follow-up, and results.

After the data were collected, the information was compared between the two reviewers. In the event of disagreements, a consensus was reached with the assistance of a third reviewer.

### Risk of bias assessment

Risk of bias assessment was applied to all the included RCTs and was based on the RoB 2 tool detailed in the Revised Cochrane risk-of-bias tool for randomized trials^[16]^.

Five distinct domains were assessed (randomization process, deviations from intended interventions, missing outcome data, measurement of the outcome, and selection of the reported result) by answering for each domain to one or more signaling questions. Then, the RoB 2 algorithm lead to a domain-level judgment risk of bias categorized as “low risk of bias,” “some concerns,” or “high risk of bias.” Then an overall risk-of-bias judgment for every study being assessed was made.

## Results

### Study selection

An overall number of 1834 publications were identified using the search method. After eliminating double entries and examining titles and abstracts, four articles were retained^[13,17–19]^. After a full text analysis, one additional article was excluded due to the fact that it was the same study but with a different follow-up. Consequently, the only study included was the one with the longest follow-up time^[18]^.

**Figure 1.**
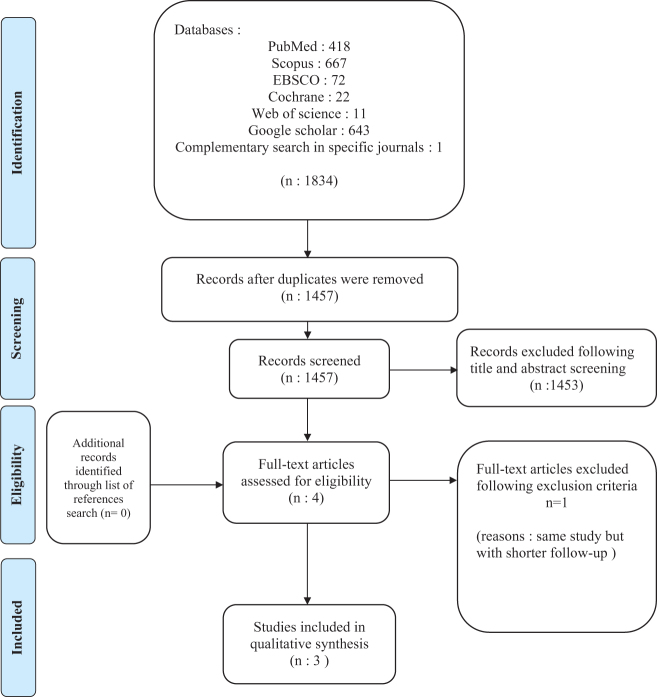
Flow-chart diagram.

At the end of the reading, three clinical studies^[17–19]^ had their eligibility confirmed. The flowchart applied during the study selection in this systematic review is outlined in Figure 1.

### Study characteristics

Three clinical trials included in this review were conducted in the same country, Iran, and were published between 2011 and 2016. The total number of participants was 108 children, ranging from 4 to 8 years old, and two of the trials justified their sample size and reported the power of their study^[17–19]^. Regarding the procedure, two of the selected studies focused on pulpotomy^[17,19]^ while the other one considered direct capping^[18]^. Follow-up periods were diverse, ranging from 7 days^[19]^ to 2 years^[17]^. Table 2 summarizes the descriptive characteristics and the main results of the selected papers (Table 2).

**Table 2:** Overview of the characteristics of included randomized clinical trials

Authors Years Country	Study design and settings	Intervention procedure	Sample characteristic	Groups	Preoperative status of the teeth	Restoration	Isolation	Assessment criteria	Follow-up period	Results
B. Malekafzali *et al*. 2011 Teheran, Iran (Malekafzali *et al*., 2011)	split-mouth randomized controlled clinical trial	Pulpotomy	40 children	CEM group: 40 teeth	Symptom-free primary molars with a deep caries lesion.	**MTA group**	Rubber dam	**clinical success or failure:**	6, 12 and 24 months	**clinical:**
			M = 23			19 (47%): SSC		*clinical failure:* if one or more of the following signs were present: swelling/abscess, sinus tract, spontaneous pain, or pathological mobility.		*At 6,12 and 24 months:*
			F =17							*100% clinical success for both group*
						6 (15%): class I A		*clinical success:* if none of these signs were present.		**radiographic:**
								**Radiographic success or failure:**		
			**N = 80 teeth**	MTA group: 40 teeth	Patients who did not have carious exposures after treatment were excluded.			*radiographic failure:* if one or more of the following signs were present: signs of furcation radiolucency, periapical bone destruction, internal root resorption, and pathological external root resorption. Arrested internal resorption with calcific metamorphosis of the pulp and pulp canal obliteration (PCO) were not regarded as failures.		*At 6 months:*
						15 (37%): class II A				*100% success for both group*
			**Age** = 6 ± 0.75 years (4 to 8 years old)							
						**CEM group:**				*At 12 months:*
								*radiographic success:* if none of these signs were present.		
						17 (42%): SSC				CEM group: *97 % success*
										MTA group: 91% success *no statistical difference between both group(P=0,625)*
						4 (10%): Class I A				
						19 (47%): Class II A				
						SSC:N=36 teeth				*At 24 months:*
										*100% success for both group for the remaining teeth (the previously failed reported at the 12months follow-up have been extracted)* there were no statistical differences between the both materials (P=0.352).
						A: N= 44 teeth				
Ghajari *et al*. 2013 Teheran, Iran (Ghajari *et al*., 2013)	split-mouth randomized controlled clinical trial	direct pulp capping	21 children	CEM group: 21 teeth	carious second primary molars were included based on symptom-free vital pulp exposure	Amalgame	cotton rolls and suction	**clinical failure:** the presence of one or more of these symptom/signs: pain, swelling, tenderness to pressure, and signs such as presence of sinus tract, swelling and tenderness to percussion.	6 and 20 months	**At 6 months**
			M = 5							
			F =16							clinical:
			**N = 42 teeth**	MTA group: 21 teeth	In cases where pulp exposures was <1 mm and bleeding arrested in <3 min, DPC were performed. The teeth with exposures >1 mm were excluded and subsequently pulpotomized .					CEM group: 94.8% success
								**radiographic failure:** internal and/or external root resorption, interradicular radiolucencies, and periapical lesions were assessed as the radiographic criteria for failure		MTA group: 100% success
			**Age** = 6.9 ± 0.7 years (5 to 8 years old)							
										radiographic:
										100% success rate for both group
										No statistical difference between the two materials (*P*=0.721)
										**At 20 months**
										CEM group: 89%
										MTA group: 95%
										without any statistical difference (*P*=0.360)
Shafie.L *et al*. 2016 Kerman, Iran (Shafie *et al*., 2017)	**Study design:** split-mouth RCT	Pulpotomy	47children	CEM group: 47 teeth	Cariously primary molars in need of pulpotomy	Stainless steel crown	Rubber dam	**Postoperative pain:** Wong-Baker faces pain rating scale (Wong- Baker FPRS)	7 days	mean (SD) of pain
			M = 17							*Day 1*
			F =28							
								**Record of the number of analgesics used (acetaminophen)**		CEM group: 2.3 +/- 1.1
				MTA group: 47 teeth						MTA group: 2.2 +/- 1.1
			**N = 94 teeth**							*Day 2*
			**Age** = 7.1±1.006 (6 to 10 years old)							
										CEM group: 1.2 +/- 0,57
										MTA group: 1.2 +/- 0,57
										*Day 3*
										CEM group: 1 +/- 0,25
										MTA group: 1+/- 0,28
										No significant difference between pain reported after using CEM cement and MTA as pulpotomy agents
										There was no significant difference between the pulpotomy agents in consumption of analgesics following treatment

A, amalgame; CEM, calcium-enriched mixture; DPC, direct pulp-capping; F: female; M, male; MTA, mineral trioxide aggregate; SSC, stainless steel crown.

### Assessment of risk of bias summary

**Figure 2.**
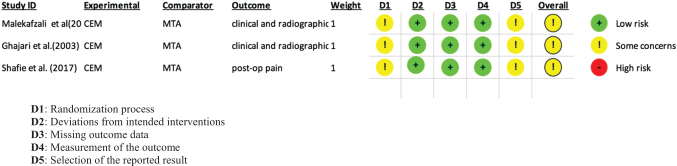
Assessment of risk of bias summary. D1: Randomization process. D2: Deviations from intended interventions. D3: Missing outcome data. D4: Measurement of the outcome. D5: Selection of the reported result.

The overall risk of bias in this systematic review raised some concerns in all the studies included (Fig. 2). The randomization process and selection of the reported results raised some concerns, while deviations from intended interventions, missing outcomes data, and measurement of the outcome were at low risk in 100% of the studies.

Concerning the randomization process, Shafie *et al*^[19]^ used a random number table for sequence generation process, while Malekafzali *et al* and Ghajari *et al*^[17,18]^ did not provide information about the randomization process. In addition, no information was given about the concealment of sequence allocation until participants were registered and assigned to interventions.

When considering the selection of the reported studies, no information was available concerning the pre-specified intentions.

## Discussion

The findings of this systematic review reveal that both CEM and MTA demonstrate comparable clinical and radiographic success when used for VPT in primary teeth. Similar results were reported by recent systematic reviews comparing the outcomes of CEM and MTA as capping material in both primary and permanent mature molars^[20]^ as well as in immature permanent teeth^[21]^.

When considering pulpotomy procedure alone in primary teeth, Malekafzali *et al* checked clinical and radiographic evaluation at 6-, 12- and 24-month recall^[17]^. Results showed that clinical and radiographic outcomes of both groups were comparable without statistically significant difference. No clinical failure was observed in either group at the 6-month, 1-year, and 2-year follow-ups. However, one case of pathological external root resorption was observed in the CEM group and three cases in the MTA group, with no significant difference between them at the 12-month follow-up. All other treated teeth in both group where symptom free at the last recall. A case report demonstrated a successful outcome 2 years after using CEM as a pulpotomy dressing material in a maxillary first primary molar. Histological examination revealed complete dentine bridge formation with regular tubular dentine, no tunnel defect, and normal underlying pulp^[22]^, which may indicate the material’s potential to promote effective dentinogenesis and maintain pulp vitality.

When performing direct pulp capping, similar result were found by Ghajari *et al* with favorable clinical and radiographic outcomes at 20 months follow-up that were similar between CEM and MTA without any statistically significant difference^[18]^. At 6 months, one of the teeth in the CEM group showed a sinus tract, while the MTA group showed no clinical signs of failure. Radiographically, there were no signs of failure in either group at 6 months. By the end of the follow-up period, one additional tooth in each group failed, with no statistical difference between them.

Zarrabi *et al* studied the histological reaction of dental pulps to MTA and CEM and found that CEM cement caused less pulpal inflammation and a thicker dentinal bridge, but these differences were not statistically significant^[10]^. However, this study was performed on healthy pulps. Therefore, these results do not necessarily reflect what will happen if they are used on reversibly inflamed pulps.

With regard to pain scores and analgesic intake, no significant differences were noted after pulpotomy using both cements in primary molars^[19]^. Similar results have also been reported for permanent teeth^[23]^.

Besides its use in VPT, CEM has demonstrated effectiveness in treating internal and external root resorption^[11,24]^, repairing furcal perforations^[25,26]^, managing open-aperture teeth^[27]^, and as a root filling material^[12]^.

Biocompatibility, non-toxicity, and antibacterial properties are the main essential characteristics of material that comes into contact with the remaining pulp^[28]^. In this regard, both cements demonstrated favorable cell viability on different cells lines^[29,30]^, and their cytotoxic potentials are both insignificant and comparable^[31,32]^. Furthermore, several studies have assessed the antibacterial properties of CEM cement against the most common endodontic pathogens. The *in vitro* study of Asgary indicated that the antibacterial activity of CEM cement against *Streptococcus mutans, Escherichia coli, Actinomyces*, and *Enterococcus faecalis* is superior to that of MTA and similar to that of calcium hydroxide^[33]^. On the contrary, the results reported by Razmi *et al* revealed the similarity of antibacterial activity against *E. faecalis* for both materials^[34]^, while Zarrabi *et al* reported ineffectiveness of both materials against *E. faecalis*^[35]^. Hence, the comparison among studies with different methodologies seems improper, and further studies with standardized protocol are needed to reach a conclusion.

From a clinical application perspective, it has been suggested that the CEM mixture is non-sticky, allowing for easy application by practitioners, and it has a shorter setting time compared to MTA. However, this shorter setting time (approximately 50 min)^[36,37]^ has limited clinical impact as it is still not suitable for a single-visit treatment. Besides, when considering potential tooth discoloration, CEM did not show any discoloration properties^[38,39]^.

As far as we are aware, this is the first systematic review to compare the performance of CEM to MTA in VPT for primary teeth. It is a relevant and a routinely procedure in pediatric practice, when well indicated, since its respects minimal intervention principles through vitality maintenance and avoid early exodontia, hence resulting in an improvement in children’s quality of life^[1]^. In our review, MTA was selected as a comparator because the highest level of evidence supports it use as a pulp capping material which may have higher clinical impact. Our study was conducted in accordance with the PRISMA guidelines for transparent reporting of systematic review and meta-analysis, ensuring that the entire process can be considered reliable^[15]^. A meta-analysis could not be performed because only two studies met the criteria for quantitative synthesis. Although the calculated *I*^2^ was approximately 40%, suggesting moderate heterogeneity, this estimate is unstable given the small number of studies. Furthermore, the wide 95% confidence interval of the pooled odds ratio indicates substantial imprecision and uncertainty. Consequently, a meta-analytic summary is methodologically unjustified, and a narrative synthesis was performed.

It is important to add that factors related to the success rate of vital pulp procedure does not depend only on the capping material used, but it also include an accurate preoperative diagnosis^[1]^, a careful procedure to avoid contamination of pulp tissue (good isolation of surgical field, removing all caries prior to opening the pulp chamber, rigorous restoration)^[40,41]^. All these factors were optimized in all our included study, which may limit the amount of confusion.

However, the results of this review should be interpreted with caution, as it includes findings from three randomized clinical trials that exhibit some inconsistencies in the randomization process and the selection of reported outcomes. The Cochrane Risk of Bias 2 tool indicated that all studies included in this systematic review raised some concerns regarding the overall risk of bias. In addition, all the selected articles originate from the same country (Iran) and two out of three articles are from the same university. Moreover, they did not assess the patient and the practitioner comfort and the handling and treatment cost (required time, materials, price), which are all important parameters in pediatric dentistry. It is also important to mention that an effective therapy in primary teeth must not only preserve the treated tooth, but it must also promote an environment conducive to its natural exfoliation, taking care not to harm or interfere with the development of the permanent successor; therefore, it would be desirable that upcoming studies with longer follow-up that covers the permutation stage include these criteria.

## Conclusion

Within the limitations of this review, CEM could be an alternative to MTA for VPT in primary teeth. However, further randomized clinical trials with larger sample sizes and extended follow-up periods are required.

## Data Availability

Data sharing is not applicable to this article as no new datasets were generated or analyzed during this study.

## Research registration unique identifying number (UIN)

Accreditation number in the PROSPERO database: CRD42021251374.
